# Isotopically non-stationary metabolic flux analysis of heterotrophic *Arabidopsis thaliana* cell cultures

**DOI:** 10.3389/fpls.2022.1049559

**Published:** 2023-01-09

**Authors:** Edward N. Smith, R. George Ratcliffe, Nicholas J. Kruger

**Affiliations:** ^1^ Molecular Plant Biology, Department of Biology, University of Oxford, Oxford, United Kingdom; ^2^ Molecular Systems Biology, Groningen Biomolecular Sciences and Biotechnology Institute, University of Groningen, Groningen, Netherlands

**Keywords:** ^13^C labelling time-course, heterotrophic carbon metabolism, INST-MFA, metabolic flux analysis, metabolic phenotype

## Abstract

Fluxes are the ultimate phenotype of metabolism and their accurate quantification is fundamental to any understanding of metabolic networks. Steady state metabolic flux analysis has been the method of choice for quantifying fluxes in heterotrophic cells, but it is unable to measure fluxes during short-lived metabolic states, such as a transient oxidative load. Isotopically non-stationary metabolic flux analysis (INST-MFA) can be performed over shorter timescales (minutes – hours) and might overcome this limitation. INST-MFA has recently been applied to photosynthesising leaves, but agriculturally important tissues such as roots and storage organs, or plants during the night are heterotrophic. Here we outline the application of INST-MFA to heterotrophic plant cells. Using INST-MFA we were able to identify changes in the fluxes supported by phosphoenolpyruvate carboxylase and malic enzyme under oxidative load, highlighting the potential of INST-MFA to measure fluxes during short-lived metabolic states. We discuss the challenges in applying INST-MFA, and highlight further development required before it can be routinely used to quantify fluxes in heterotrophic plant cells.

## Introduction

Steady-state metabolic flux analysis (SS-MFA) is a well-established method for measuring multiple fluxes in plant metabolic networks (Ratcliffe and Shachar-Hill, 2006; Kruger and Ratcliffe, 2015; Moreira et al., 2019; Allen and Young, 2020). Typically, fluxes are deduced from the steady state labelling patterns observed when a tissue in a metabolic steady state is incubated with a ^13^C-labelled substrate. Although this method has been used extensively to characterise the metabolic phenotypes of heterotrophic and mixotrophic plant systems, including arabidopsis cell cultures (Masakapalli et al., 2013; Masakapalli et al., 2014) and cultured oil seed embryos (Schwender et al., 2015; Carey et al., 2020), the need to establish an isotopic steady state while maintaining a metabolic steady state limits the range of applications.

Even when metabolic and isotopic steady states can be achieved, a major disadvantage of SS-MFA is that it cannot be used to probe photosynthetic metabolism, because steady-state labelling with ^13^C-labelled carbon dioxide results in a uniform, and therefore uninformative, labelling of every metabolite in the network (Roscher et al., 2000). The solution is to analyse labelling time-courses, rather than the isotopic steady state that is eventually achieved, and this is the basis of the technique known as isotopically non-stationary metabolic flux analysis (INST-MFA) (Wiechert and Nöh, 2013; Cheah and Young, 2018). Although challenging to implement, the development of the method for studying light-dependent metabolism in cyanobacteria (Young et al., 2011) has been followed by several applications to autotrophic metabolism in leaves (Ma et al., 2014; Xu et al., 2021; Chu et al., 2022; Treves et al., 2022; Xu et al., 2022).

The success of INST-MFA in analysing plant autotrophic metabolism raises the question of whether it could also be useful in extending the analysis of heterotrophic metabolic phenotypes. The SS-MFA approach requires the system to be in both isotopic and metabolic steady state making it difficult to analyse the metabolic response to physiological perturbations. For example, SS-MFA cannot be used to investigate perturbations arising from the imposition of hypoxia or oxidative stress, because the metabolic state of the system is likely to change faster than the time taken to reach an isotopic steady state during a labelling experiment. While it may take days to reach an isotopic steady state in the end-products of metabolism (Kruger et al., 2012), INST-MFA relies on measurements of labelling time-courses of intermediates over a period of minutes to hours, making it potentially more suitable for the analysis of metabolism in a system where the metabolic steady state is changing or short-lived.

To test the practicality of this proposal, this study reports the application of INST-MFA to an arabidopsis cell culture undergoing the mild oxidative stress induced by exposure to menadione. The arabidopsis cell culture has been used as a model system for SS-MFA studies of heterotrophic metabolism (Kruger et al., 2012) and the effect of menadione on the cells is also well characterised (Baxter et al., 2007). The results highlight the experimental challenges in applying INST-MFA to plant cell cultures and leads to the conclusion that further development is required before the technique can match the utility of SS-MFA.

## Materials and methods

### Arabidopsis cell cultures

Cell suspensions of *Arabidopsis thaliana* (ecotype Landsberg *erecta*) were cultured in Murashige and Skoog (MS) medium at pH 5.8 containing 30 g/L glucose, 0.5 mg/L 1-naphthaleneacetic acid, and 0.05 mg/L kinetin (Williams et al., 2008). Cultures were grown in darkness at 22 °C in sterile 250 mL Erlenmeyer flasks, sealed with a double layer of aluminium foil, on an orbital shaker running at 120 rpm. The cell line was maintained by weekly subculturing of 15 mL of cell culture into 100 mL of fresh medium.

### Reactive oxygen species assay

Liquid cell cultures were assayed by incubating 4-d-old cell suspension culture with 60 µM or 200 µM menadione, or ethanol (EtOH) control, for 6 h before adding 2.5 µM 2’,7’-dichlorodihydrofluorescein diacetate (H_2_-DCFDA). End point fluorescence was measured (excitation 504 nm, emission 529 nm) after 1.5 h. Background fluorescence was monitored using cells incubated without H_2_-DCFDA and by measuring the fluorescence of the dye in the presence of menadione and MS medium without cells.

### [^13^C_6_]glucose labelling

Menadione dissolved in EtOH was added to 115 mL of 4-d-old heterotrophic arabidopsis cell cultures to a final concentration of 60 µM (0.065% EtOH v/v) and incubated for 6 h in the dark. A cell sample was taken before the addition of labelled glucose (0 min). [^13^C_6_]glucose dissolved in MS medium was added to give a fractional enrichment of ~60% and a final concentration of ~120 mM glucose. The amount of labelled glucose to add was calculated based on the glucose concentration of the medium immediately prior to labelling. Cells were incubated in the dark and samples taken at 0, 0.5, 1, 2, 4, 8, 10, 15, 20, 30, 60, 120, and 270 min after addition of labelled substrate. Care was taken to minimise light exposure by using minimal green lighting.

### Extraction of liquid cell cultures

Five ml of 4-d-old arabidopsis cell culture (~300 – 500 mg fresh weight) was rapidly filtered under vacuum (<10 s) on to Whatman No. 1 filter paper (42.5 mm diameter) and the cells were scraped into 1.35 mL of 2:1 (by volume) dichloromethane:ethanol kept on dry ice (**−**78 °C). Subsequently, 150 µL of 60 mM HCl was added and the mixture was vortexed for 10 s before centrifugation at 20,000 x g for 10 min (4 °C). A 400 µL aliquot of the resulting aqueous phase was stored on ice and adjusted to below pH 2.5 by addition of 45 µL 0.48 M HCl. Each sample was then filtered using a 10 kDa MWCO filter (Amicon), transferred to a glass LC-MS vial and stored at **−**80 °C before analysis by LC-MS.

### Liquid chromatography mass spectrometry analysis of cell extracts

Metabolite extracts were analysed using a Thermo Scientific ICS-5000+ ion chromatography system coupled directly to a Q-Exactive Hybrid Quadrupole-Orbitrap mass spectrometer with a HESI II electrospray ionization source (Thermo Scientific, San Jose, CA, USA) (Walsby-Tickle et al., 2020). The ICS-5000+ HPLC system incorporated an electrolytic anion generator (KOH) which was programmed to produce a 5–100 mM OH-gradient over 37 min to facilitate ion chromatography. An inline electrolytic suppressor removed hydroxide ions and cations from the post-column eluent prior to MS analysis (Thermo Scientific Dionex AERS 500). A 10 μL partial loop injection was used for all analyses and the chromatographic separation was performed using a Thermo Scientific Dionex IonPac AS11-HC 2 × 250 mm, 4 μm particle size column with a Dionex Ionpac AG11-HC 4 μm 2 × 50 guard column inline. The flow rate was 0.25 mL/min. The total run time was 37 min and the hydroxide ion gradient was: 0 min, 0 mM; 1 min, 0 mM; 15 min, 60 mM; 25 min, 100 mM; 30 min, 100 mM; 30.1 min, 0 mM; 37 min, 0 mM. Analysis was performed in negative ion mode using a scan range from 60 to 900 and resolution set to 70,000. The tune file source parameters were: sheath gas flow 60 mL/min; auxiliary gas flow 20 mL/min; spray voltage 3.6 V; capillary temperature 320 °C; S-lens RF value 70; heater temperature 450 °C. Automatic gain control target was set to 1×10^6^ and the maximum injection time value was 250 ms. The column temperature was maintained at 30 °C.

### Mass isotopologue distribution measurement and natural abundance correction

LC-MS data were analysed using El-Maven software (Agrawal et al., 2019) and compounds identified using an in-house database of authentic standards (Walsby-Tickle et al., 2020). Mass isotopologue distributions (MIDs) were corrected for natural abundance of heavy isotopes using AccuCor (Su et al., 2017) (**Supplementary Data 1**). The MID of UDP was deconvoluted from the MID of UDP-glucose using least squares fitting implemented in MATLAB (see https://github.com/tednsmith/heterotrophic_INST-MFA for code). Fractional atom enrichment is the probability that any carbon atom is isotopically labelled and was calculated by the equation $\sum^{\text{n}}\text{i}{=}_{1}(a_{i}.i)/n$ where *a*_*i*_ is the fractional abundance of isotopologues containing *i* ^13^C atoms and *n* is the total number of carbon atoms in the molecule.

### Model construction and flux estimation

Metabolic modelling was performed using the Isotopomer Network Compartmental Analysis software package (INCA v1.9, http://mfa.vueinnovations.com, Vanderbilt University) (Young, 2014) implemented in MATLAB 2019b (MathWorks, Natick, Mass, USA). A network of carbon atom transitions was defined based on the model of central carbon metabolism in heterotrophic arabidopsis cell cultures used previously (Masakapalli et al., 2014). The full model definition can be found in **Supplementary Data 2** and online at https://github.com/tednsmith/heterotrophic_INST-MFA. Pseudo fluxes were added to describe mixing of subcellular pools of metabolites upon extraction, or mixing of pre-existing unlabelled pools of certain metabolites (Kruger et al., 2014). Fluxes were estimated using a Levenberg-Marquardt optimization algorithm (Levenberg, 1944) to minimize the differences between the measured and simulated mass isotopologue distributions by minimising the sum of squared residuals (SSR) weighted by the individual measurement variance (Young, 2014). A fit is accepted if the SSR falls within a range defined by the equation χα22(n−p), χ1−α22(n−p) where *n* is the number of independent measurements, *p* is the number of fitted parameters and (*n* – *p*) is the degrees of freedom. Free parameters were iteratively updated to identify a best-fit solution. Analyses were repeated 32 times, each from a random set of starting values to avoid local minima and identify a global best fit. Consistent errors for each measured mass isotopologue at each time point within each experiment were determined by systematically varying the error and recalculating the SSR. Mol% errors were set to a minimum value for low abundance isotopologues (≤ 0.5 mol%) and scaled linearly up to a maximum error for higher abundance isotopologues (≥ 25 mol%) (Young, 2014). See **Table 1** for the error ranges used. The scaling of errors for isotopologue abundances ≤ 25 mol% is to avoid setting overly large errors on small isotopologue measurements. Varying the errors in the MID measurements allows a statistically acceptable fit to be achieved at the cost of precision in flux estimates. Assuming the model definition is sufficient to describe the data, this avoids biasing the flux estimates to overly precise values.

**Table 1:** Summary of characteristics of best-fit flux models for control cells and cells treated with 60 µM menadione.

Model parameter	Control	Menadione
Isotopologue measurements	4418	4218
Measurement error (mol%)	1.20 – 1.90	1.45 – 2.15
Flux measurements	30	30
Total free parameters	847	815
MS normalisations	698	666
Number of free fluxes	96	96
Number of free pool sizes	53	53
Degrees of freedom	3601	3433
Total residuum (SSR)	3758.0	3353.0
Critical χ^2^ value (p = 0.05)	3436.6 – 3769.2	3272.5 – 3597.3

Each model was simultaneously fitted to labelling datasets from three independent cell cultures. Measurement errors were set to a minimum value for MIDs ≤ 0.5mol% and scaled linearly up to a maximum error for MIDs ≥ 25 mol% (Young, 2014). Flux measurements were defined as biomass outputs from Masakapalli et al. (2014). MS normalisations refer to the scaling factors applied to mass isotopologues to account for any missing or anomalous mass isotopologue measurements (Young, 2014)

### Flux map statistical analysis

Two methods were used to estimate the precision of the deduced fluxes, parameter continuation and Monte Carlo simulation. Parameter continuation was performed using the ‘continuate’ function in INCA to generate the 95% confidence interval for each parameter. This function measures the sensitivity of the SSR to variation in each parameter individually (Antoniewicz et al., 2006). Any improvements in the best-fit solution identified during parameter continuation were retained in subsequent analyses and the parameter continuation repeated. Monte Carlo simulation was performed both to determine parameter uncertainty and to compare the probability distributions of parameter estimates between control and menadione-treated cells. To perform Monte Carlo simulations, the best-fit set of parameters was used to generate multiple synthetic datasets which were perturbed by Gaussian noise based on the measurement errors. Each synthetic dataset was then refitted to generate a set of optimal parameters. Repeating this process many times generated a probability distribution for each parameter. 1000 rounds of Monte Carlo simulation were run using the ‘montecarlo’ function in INCA based on the method described by Press and Vetterling (1992) (Young, 2014).

Significant differences between parameters in control and menadione flux maps were calculated by comparing the 83.4% confidence intervals, which is equivalent to a significance of P = 0.05 assuming that variance between the two groups is equal (Austin and Hux, 2002; Knol et al., 2011). For any parameters with unequal variances, the confidence intervals to compare were calculated by the equation $\left[1-2\times \Phi \left(-1.96\times \frac{\sqrt{1+\rho^{2}}}{1+\rho }\right)\right]\times 100\%$ where *ρ* represents the ratio of standard deviations and Φ represents the standard normal cumulative distribution function (Knol et al., 2011). The confidence intervals were calculated from Monte Carlo simulations by ranking the parameter estimates in ascending order and discarding the appropriate number of values from the top and bottom of the range. For example, the top and bottom 8.3% of values are discarded for an 83.4% confidence interval and the top and bottom 2.5% values for the 95% confidence interval.

Principal components analysis (PCA) and partial least squares discriminant analysis (PLSDA) were performed on 1000 Monte Carlo simulations for control and menadione-treated cells using MetaboAnalyst, an online implementation of R for metabolomics (Chong et al., 2019; R Core Team, 2020). Net fluxes were mean centred and scaled to unit variance prior to PCA and PLSDA. Pool size estimates, exchange fluxes and pseudo fluxes describing the mixing of compartmented or metabolically inactive pools of metabolites were excluded from the analysis. PCA was performed using the ‘prcomp’ package in R (R Core Team, 2020). Partial least squares-discriminant analysis (PLSDA) was performed using the ‘pls’ package in R (Mevik and Wehrens, 2007). See **Supplementary Figures S7**–**S10** for further details and validation of PLSDA.

### High performance parallel computation

INST-MFA is computationally intensive and therefore parameter estimation, parameter continuation and Monte Carlo simulation were performed using a parallel processing cluster at the University of Oxford Advanced Research Computing (ARC) facility. Parallel jobs were submitted using the SLURM job scheduler. The parallel job submission script was modified from the implementation described in Xu et al. (2021).

## Results

### Menadione treatment induced oxidative load

**Figure 1:**
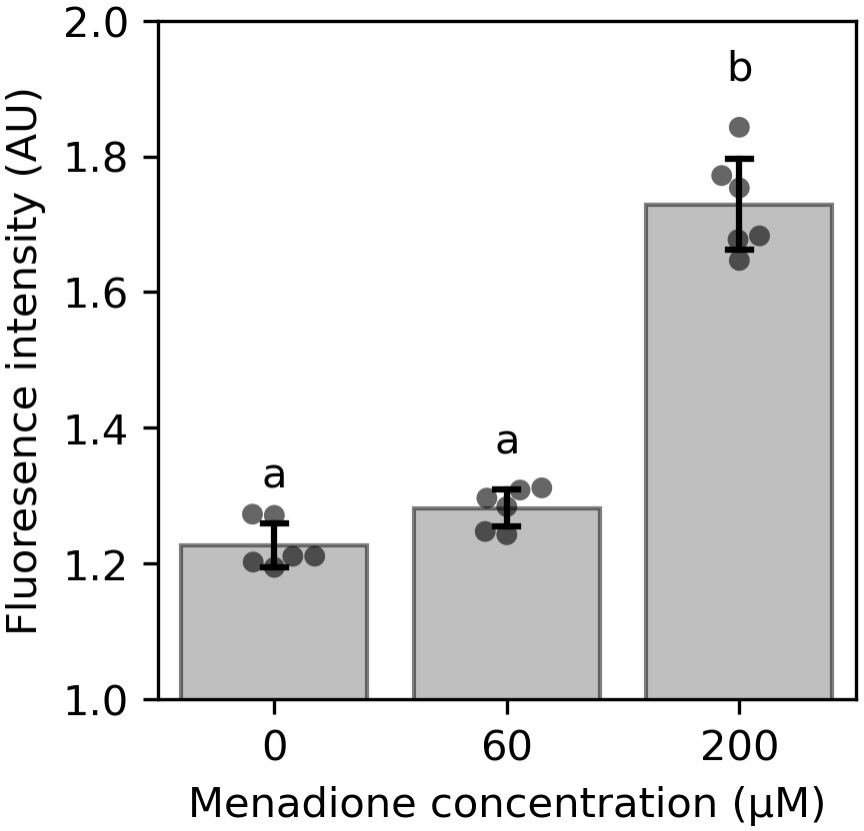
Effect of menadione on ROS production in 4-d-old heterotrophic arabidopsis cell cultures quantified by H_2_-DCFDA fluorescence. Cells were incubated with menadione for 6 h prior to measurement. Values are the mean ± SD, n = 6. Different letters represent values that differ significantly as determined by one-way ANOVA followed by Tukey test (P< 0.05). AU – arbitrary units.

Cells were incubated with 60 µM or 200 µM menadione for 6 h and production of reactive oxygen species (ROS) was quantified using H_2_-DCFDA (**Figure 1**). Incubation of cell cultures with 200 µM menadione for 6 h caused a significant increase in H_2_-DCFDA fluorescence (**Figure 1**). In contrast, 60 µM menadione had no effect on H_2_-DCFDA fluorescence compared to an EtOH treated control. These results indicate that although menadione stimulates ROS production, the oxidative challenge imposed by 60 µM menadione does not overwhelm the capacity of the cellular ROS detoxifying systems, and accordingly this concentration was used for subsequent labelling experiments.

### ^13^C-Labelling of heterotrophic arabidopsis cells treated with menadione

**Figure 2:**
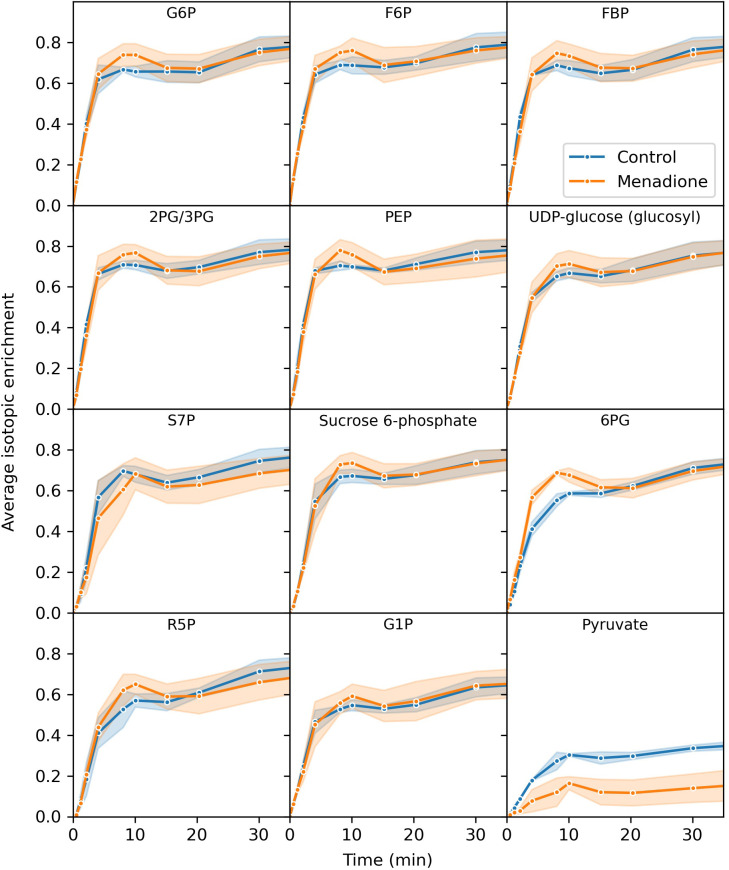
Average isotopic enrichment of glycolytic, pentose phosphate pathway and starch/sucrose synthesis intermediates after supply of [^13^C_6_]glucose to heterotrophic arabidopsis cell cultures following 6 h treatment with 60 µM menadione or untreated control. Isotopic enrichment is corrected for natural abundance of heavy isotopes and the proportion of labelled substrate in the media. Values are the mean ± SD (shaded region), n = 3.

**Figure 3:**
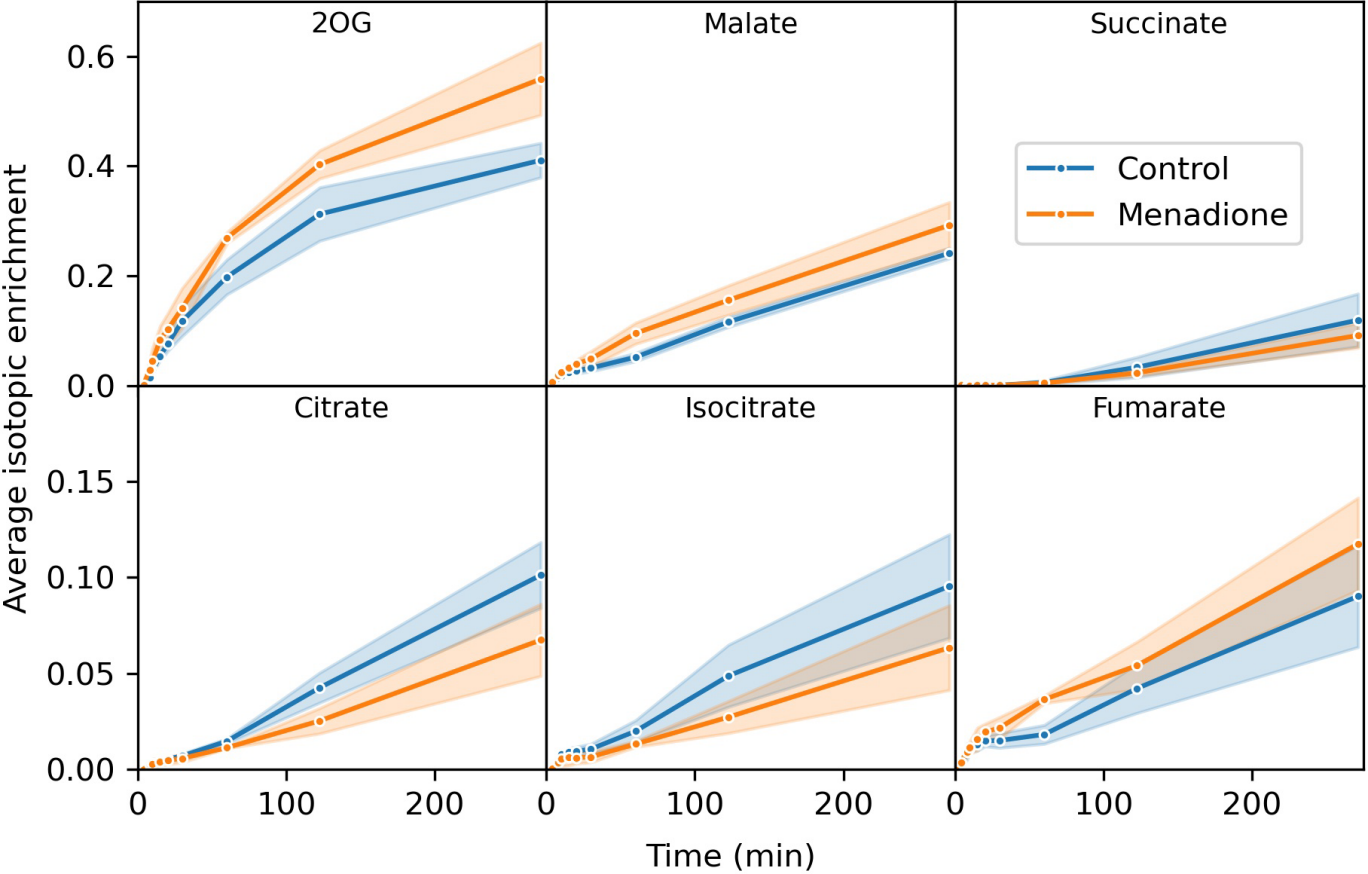
Average isotopic enrichment of TCA cycle intermediates after supply of [^13^C_6_]glucose to heterotrophic arabidopsis cell cultures following 6 h treatment with 60 µM menadione or untreated control. Isotopic enrichment is corrected for natural abundance of heavy isotopes and the proportion of labelled substrate in the media. Note the difference in scales on the y-axis in different panels. Values are the mean ± SD (shaded region), n = 3.

Three replicate flasks of heterotrophic arabidopsis cells treated with 60 µM menadione for 6 h, or an untreated control, were labelled with a bolus of [^13^C_6_]glucose, resulting in approximately 60% labelling of glucose in the media (**Table S1**). Immediately after addition of ^13^C-glucose, cell samples were extracted at 13 time points over 4.5 h and the mass spectra of 18 metabolites analysed by LC-MS. The average isotopic enrichment of each metabolite increased over time, with glycolytic intermediates labelled fastest followed by those of the pentose phosphate pathway (PPP) and starch/sucrose synthesis, with tricarboxylic acid (TCA) cycle intermediates labelled the slowest (**Figures 2**, **3**, **S1**–**S3**). 2-Oxoglutarate was labelled more rapidly than citrate and isocitrate, as well as other TCA cycle intermediates, indicating the presence of metabolically less-active pools of these organic acids (**Figure 3**). The average isotopic enrichment did not reach the isotopic enrichment of the supplied substrate for any metabolite suggesting the presence of unlabelled pools of metabolites. These pools could either be metabolically active and provide an input of unlabelled carbon into the system, or metabolically inactive and simply dilute the labelled pools of metabolites upon extraction. The vacuole likely contains relatively large pools of metabolites, including sugars which could be metabolised over the time-course of the labelling experiment (Szecowka et al., 2013; Xu et al., 2022). Interestingly, there was a small decrease in average isotopic enrichment of phosphate esters between 4 and 15 min, again consistent with input of carbon from unlabelled pools of carbohydrates.

The extent of labelling for many metabolites was largely unaffected by menadione treatment with the exception of pyruvate and 2-oxoglutarate. Labelling of pyruvate was decreased by menadione treatment despite labelling of the immediate precursor, phosphoenolpyruvate (PEP), being unaffected. 2-Oxoglutarate was labelled more rapidly in the presence of menadione. Overall, these features justified the use of a model reflecting the conventional pathways of central carbon metabolism; glycolysis, the PPP, and TCA cycle with withdrawal of intermediates for production of sucrose, starch and amino acids.

It was observed that arabidopsis cultures exist as aggregates of cells in suspension (**Figure S4**) and therefore the potential effect of limited diffusion of labelled substrate to cells was simulated (**Figure S5**). Comparison of the simulated labelling data to the observed labelling of glucose 6-phosphate (the immediate product of imported glucose) suggests that cell aggregation had a negligible effect on the labelling time-course of metabolic intermediates (**Figures 2**, **S5**). The effects of delayed substrate supply on the SSR for an idealized dataset were also simulated and showed that there was only a small increase in the SSR (<10) for delays in substrate arrival of up to 5 min (**Figure S5**).

### The effect of menadione treatment on heterotrophic cell cultures

**Figure 4:**
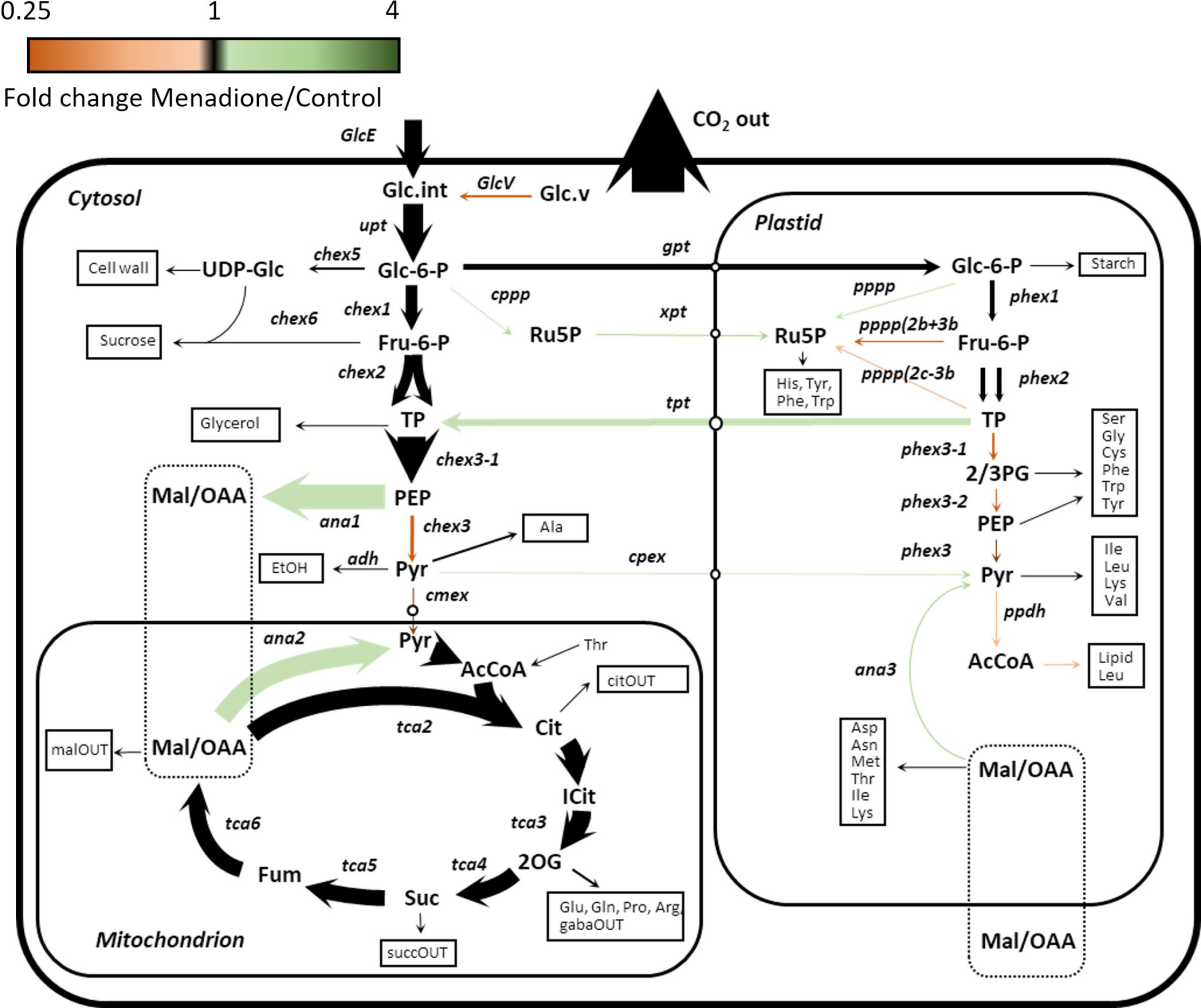
Flux map of central carbon metabolism in heterotrophic arabidopsis cell cultures treated with 60 µM menadione for 6 h or untreated control. Fluxes were deduced using INST-MFA following supply of [^13^C_6_]glucose. The arrow thickness is proportional to relative molar flux in menadione-treated cells. Arrow colour reflects the ratio of fluxes between menadione-treated and control cells. Numerical values of all fluxes are presented separately (**Table 2**, **Supplementary Data**).

A model of mass isotopomer interconversions was fitted to the experimental labelling data in combination with published output fluxes based on [U-^14^C]glucose labelling and biomass composition analysis of an identical cell culture (Masakapalli et al., 2014). Uncertainties in isotopologue measurements were scaled to accommodate the experimental and biological variability in order to achieve statistically acceptable fits of the model to the data (**Figure S6**). A greater uncertainty was required in menadione-treated cells to achieve an acceptable SSR value (**Table 1**). Monte Carlo simulations were performed by sampling the labelling time-courses and overlap between confidence intervals used to identify significantly different fluxes. Multivariate statistics using PCA and PLSDA was also used to identify fluxes that differed significantly between the two conditions (**Figures S7**, **8**). These differences were limited to a decrease in flux through pyruvate kinase (chex3) and an increase in flux through PEP carboxylase (ana1) and malic enzyme (ana2) in response to menadione treatment (**Figure 4** and **Table 2**). Input of unlabelled carbon (GlcV) significantly decreased in menadione-treated cells either as a result of an increased uptake of glucose from the medium, or a decrease in the mobilization of unlabelled stored carbon. Metabolite pool sizes were also determined as an output of the INST-MFA fitting procedure (**Supplementary Data 2**). However, when MID measurements alone are used to determine both fluxes and pool sizes, the resulting pool sizes determined by the fitting procedure can differ substantially from accurate values, and therefore they were not interpreted further (Zheng et al., 2022). Overall, these comparisons suggest that treatment with 60 µM menadione caused only relatively subtle changes in flux through the network of central carbon metabolism and did not result in a marked perturbation of cellular functions.

**Table 2:** Global best-fit flux estimates for heterotrophic arabidopsis cell cultures treated with 60 µM menadione for 6 h or an untreated control. Fluxes were deduced using INST-MFA following supply of [^13^C_6_]glucose.

Flux	Equation	Relative molar flux (95% confidence interval)			
		Control		Menadione	
*Hexose/triose phosphate metabolism*					
upt	GLC.int -> G6P.c	1.000	*(1.000-1.000)*	1.000	*(1.000-1.000)*
**Glcv**	**GLC.v -> GLC.int**	**0.162**	***(0.156-0.167)***	**0.113**	***(0.102-0.122)***
chex1	G6P.c<-> F6P.c	0.599	*(0.555-0.646)*	0.587	*(0.533-0.654)*
chex2	FBP.c<-> TP.c + TP.c	0.540	*(0.486-0.606)*	0.529	*(0.460-0.608)*
chex3-1	TP.c<-> 3PG.c	1.315	*(1.210-1.540)*	1.417	*(1.267-1.559)*
**chex3**	**PEP.c -> PYR.c**	**0.477**	***(0.380-0.633)***	**0.173**	***(0.09-0.342)***
phex1	G6P.p<-> F6P.p	0.237	*(0.199-0.279)*	0.246	*(0.182-0.291)*
phex2	FBP.p<-> TP.p + TP.p	0.233	*(0.196-0.274)*	0.243	*(0.184-0.291)*
phex3-1	TP.p<-> 3PG.p	0.222	*(0.067-0.249)*	0.119	*(0.039-0.181)*
phex3-2	3PG.p<-> PEP.p	0.145	*(0.02-0.176)*	0.043	*(0.03-0.106)*
phex3	PEP.p -> PYR.p	0.121	*(0.000-0.151)*	0.020	*(0.008-0.082)*
*Pentose phosphate pathway*					
cppp1	G6P.c -> 6PG.c	0.000	*(0.000-0.000)*	0.000	*(0-0.01)*
pppp1	G6P.p -> 6PG.p	0.004	*(0.002-0.022)*	0.007	*(0.004-0.043)*
pppp2a	R5P.p + TKC2.p<-> S7P.p	0.004	*(0.002-0.010)*	0.005	*(0.003-0.018)*
pppp2b	E4P.p + TKC2.p<-> F6P.p	-0.008	*(-0.011-0.010)*	-0.007	*(-0.009-0.018)*
pppp2c	X5P.p<-> TP.p + TKC2.p	-0.004	*(-0.007-0.008)*	-0.002	*(-0.004-0.025)*
pppp3a	S7P.p<-> E4P.p + TAC3.p	0.004	*(0.002-0.01)*	0.005	*(0.003-0.018)*
pppp3b	TP.p + TAC3.p<-> F6P.p	0.004	*(0.002-0.010)*	0.005	*(0.003-0.018)*
*Transporters/exchanges*					
**cmex**	**PYR.c -> PYR.m**	**0.373**	***(0.257-0.428)***	**0.000**	***(0-0.191)***
cpex	PYR.c -> PYR.p	0.000	*(0.000-0.143)*	0.070	*(0-0.109)*
gpt	G6P.c<-> G6P.p	0.303	*(0.259-0.346)*	0.315	*(0.25-0.367)*
tpt	TP.c<-> TP.p	-0.239	*(-0.428-0.169)*	-0.363	*(-0.482–0.226)*
*Tricarboxylic acid cycle*					
tca1	PYR.m -> CO2 + AcCoA	0.998	*(0.862-1.131)*	1.008	*(0.835-1.13)*
tca2	AcCoA + MAL -> CIT	0.972	*(0.858-1.111)*	0.982	*(0.842-1.144)*
tca3	ICIT -> CO2 + 2OG	0.943	*(0.829-1.083)*	0.953	*(0.812-1.115)*
tca4	2OG -> CO2 + SUCCCoA	0.811	*(0.695-0.953)*	0.821	*(0.68-0.982)*
tca5	SUCC<-> FUM	0.801	*(0.684-0.941)*	0.811	*(0.67-0.976)*
tca6	FUM<-> MAL	0.804	*(0.687-0.944)*	0.814	*(0.672-0.98)*
*Anaplerotic fluxes*					
**ana1**	**PEP.c + CO2<-> MAL**	**0.838**	*(0.769-1.026)*	**1.243**	*(1.001-1.401)*
**ana2**	**MAL -> PYR.m + CO2**	**0.625**	*(0.541-0.769)*	**1.008**	*(0.741-1.116)*
ana3	MAL -> PYR.p + CO2	0.000	*(0-0.076)*	0.020	*(0.007-0.074)*
*Output fluxes*					
CO2out	CO2 -> CO2.eff	2.678	*(2.306-3.078)*	2.698	*(2.273-3.101)*
*Starch synthesis*					
phex4/5	G6P.p<-> G1P.p	0.061	*(0.032-0.089)*	0.063	*(0.033-0.092)*
*Sucrose synthesis*					
chex4/5	G6P.c<-> G1P.c	0.098	*(0.068-0.125)*	0.097	*(0.067-0.13)*
chex6	F6P.c + UDPG -> S6P	0.059	*(0.03-0.085)*	0.058	*(0.028-0.09)*

Values are the molar flux relative to glucose uptake. The 95% confidence intervals (italics) of free fluxes were determined by Monte Carlo simulation (lower bound – upper bound). Bold values indicate a significant difference (P< 0.05, non-overlapping 83.4% confidence intervals calculated by Monte Carlo simulation). See **Supplementary Data** for the complete set of flux values and reaction definitions..

## Discussion

### INST-MFA resolves fluxes in heterotrophic arabidopsis cell cultures

In this study INST-MFA was able to generate well resolved flux maps of central carbon metabolism of heterotrophic plant cells using labelling information obtained from phosphate esters and related metabolic intermediates. For most reactions in the network the precision of the flux estimates is comparable to those obtained by SS-MFA of the same cell culture, involving substantially longer labelling time-courses (Masakapalli et al., 2013; 2014). Statistically acceptable flux solutions were dependent on the inclusion of three features in the metabolic model, similar to those required for SS-MFA (Kruger et al., 2014): (i) metabolically less active pools of organic acids representing vacuolar stores; (ii) inclusion of distinct compartmented pools of metabolites (as well as pseudo reactions to represent compartment mixing during metabolite extraction); and (iii) incorporation of an unlabelled input of carbon (representing turnover of starch and soluble sugars (Xu et al., 2022)). It was also necessary to scale the uncertainty in the measured MIDs in order to achieve statistically acceptable fits, as opposed to using the standard error of the individual MID measurements (**Figure S6**).

The limited differences between the flux maps obtained in the presence and absence of menadione suggest that the oxidative load imposed by 60 µM menadione was relatively small and thus accommodated by the endogenous ROS detoxification mechanisms of the cell. Nevertheless, statistically significant differences were observed in fluxes associated with the metabolism of PEP and malate/OAA demonstrating the ability of INST-MFA to resolve small, localised perturbations in flux within the metabolic network. Rearrangement of fluxes around these metabolites may be a consequence of altered NADPH demands due to the increased oxidative load. Increased flux from PEP to dicarboxylates and subsequent decarboxylation to pyruvate could proceed *via* NADP-dependent isoforms of malic enzyme which have been associated with oxidative stress responses in plants (Wheeler et al., 2005; Chen et al., 2019; Sun et al., 2019). However, the oxPPP, which would be expected be active even under control conditions carried negligible flux, despite label incorporation into 6-phosphogluconate, the first intermediate of the pathway (Garlick et al., 2002; Kruger and von Schaewen, 2003). The lack of oxPPP flux is also in contrast to the results obtained in previous SS-MFA of the same cell cultures (Masakapalli et al., 2014) and this difference should be explored in future work.

Previous analysis of heterotrophic cell cultures treated with menadione identified changes in the abundance and label incorporation of TCA cycle intermediates that were considered to be consistent with a decrease in flux through the TCA cycle (Baxter et al., 2007). In contrast, no significant differences in TCA cycle fluxes were observed in the present study, most likely because INST-MFA was performed 6 h after treatment with menadione, when a new metabolic steady state had been reached, and not immediately following treatment as analysed previously (Baxter et al., 2007). Furthermore, previous analysis identified decreased label incorporation into TCA cycle intermediates but did not quantify the label importation into the upstream metabolite pyruvate. Interestingly, a decrease in label incorporation into pyruvate was observed in this study, which may explain the changes in TCA cycle intermediate labelling observed previously (Baxter et al., 2007). Despite the differences in the timing of the measurement, the global analysis presented here highlights the importance of using a model-based approach to account for the connectivity between mass isotopomers.

### Experimental considerations

The reliability of INST-MFA as an experimental technique depends critically on the protocol used to acquire the labelling data (Cheah and Young, 2018). Labelled substrate should ideally be supplied instantaneously and homogenously to the population of cells being studied to minimise the complexity of the fitted model. Data collection needs to begin early in the labelling time-course and is therefore sensitive to any perturbation caused by supplying the labelled substrate. Exchanging growth media by filtration, centrifugation or sedimentation can cause unwanted metabolic perturbation and has previously been shown to cause a large decrease in ascorbate concentration in heterotrophic arabidopsis cells, potentially due to oxidative stress induced by the handling of the cell cultures (Baxter et al., 2007). To avoid these issues in the present study, a bolus of labelled glucose was added to the cell cultures increasing the media glucose concentration from ~60 mM to ~120 mM. Since plant plasma membrane glucose transporters have K_m_ values for glucose in the range 0.01 – 2 mM (Büttner, 2007), it is likely that glucose uptake was saturated both before and after addition of labelled glucose. However, despite these efforts to minimise any metabolic perturbation, a short-lived decrease in the average isotopic enrichment of phosphate esters was observed, as opposed to the expected smooth increase (**Figure 2**). The likely explanation for this fluctuation is an increase in the input of unlabelled carbon to the cells arising from either the addition of the bolus of labelled substrate, the change in oxygen availability as the lid of the flask was removed (Williams et al., 2008), or the change in water potential of the medium (Williams et al., 2010). These considerations highlight the difficulty of maintaining constant conditions during the supply of labelled substrate to heterotrophic tissues.

For accurate INST-MFA, labelled substrate must diffuse to all cells in the sampled population rapidly, relative to the rate of metabolite turnover. For studies of autotrophic metabolism in leaves, this is unlikely to be an issue as the labelled substrate is gaseous ^13^CO_2_. However, heterotrophic tissues rely on input of soluble carbon sources in aqueous media and therefore any limitation to diffusion of the supplied substrate must be considered. For example, heterotrophic cell cultures typically exist as cell aggregates (**Figure S4**) and other heterotrophic tissues such as roots or storage organs have tissue structures that could slow diffusion of supplied labelled substrates. Simulation of a non-uniform population of cells highlighted that even where diffusion limitations are significant, they might only have a small impact on the SSR (**Figure S5**). Therefore, delay in the arrival of labelled substrate to cells should be assessed from the labelling time-course of metabolites, not just from the SSR during fitting. In this study, analysis of the labelling time-course of glucose 6-phosphate, the metabolite immediately downstream of the labelled substrate, showed any delay in the arrival of labelled substrate was likely minimal and therefore not an issue in this cell culture system. However, this issue is particularly relevant for the application of INST-MFA to more structured heterotrophic plant tissues such as roots, and any system would need to be assessed to ensure that the rate of diffusion of labelled substrate was sufficiently rapid to have no effect on the deduced fluxes.

It is essential that metabolism is quenched rapidly after collection of the sample in order to capture the required data for INST-MFA. In general, for analysis of cells in liquid media, it is desirable to separate cells from the culture medium to allow accurate measurement of the intracellular metabolite MIDs (Millard et al., 2014). This was achieved here by rapid filtration followed by quenching and extraction in cold solvent at low pH. The resulting labelling time-courses suggest this method quenched metabolism and captured labelling dynamics accurately. However, rapid filtration may not be sufficient for sample harvesting in other systems. For example, in *E. coli*, immediate quenching in cold solvent followed by centrifugation prior to extraction gave more accurate flux estimations than rapid filtration methods (Millard et al., 2014). Direct cold solvent quenching requires validation of quenching, assessment of the extent of metabolite leakage and demonstration of effective removal of external medium during extraction, but this method has been successfully applied to INST-MFA of photosynthetic microorganisms and could be applied to heterotrophic plant tissues (Sake et al., 2019).

The deduced fluxes and their confidence intervals are critically dependent on the quality of the input labelling data. However, determining the reliability of these data can be problematic (Sundqvist et al., 2022). Here the use of standard deviations calculated from the three replicate samples did not provide statistically acceptable fits. These standard deviations varied between time points and so introduced unequal weighting to different time points, not necessarily reflecting the true degree of uncertainty. To resolve this issue, consistent uncertainties across time points were applied to the labelling data in order to achieve statistically acceptable fits (**Table 1**, **Figure S6**). The uncertainty in the underlying input data is reflected in the precision of estimated fluxes and therefore avoids biasing the estimated fluxes, assuming the model is sufficient to accurately describe the data.

Pool size measurements can potentially improve the precision of flux estimates from INST-MFA but are not strictly required (Adebiyi et al., 2015; Zheng et al., 2022). Subcellular metabolite concentrations are difficult to quantify in a compartmented plant cell, and they were not used here. Furthermore, inclusion of pool size measurements can potentially lead to less accurate flux estimates if the fitted model omits reactions that interact with the measured metabolites (Zheng et al., 2022). Therefore the inclusion of pool size measurements should only be considered for accurately measured metabolites, and when the fitted model is sufficiently complete (Zheng et al., 2022), under which circumstances more accurate flux estimates can be obtained (Nöh et al., 2007; Heise et al., 2015).

Overall, the methods described here were sufficient to resolve fluxes in a heterotrophic arabidopsis cell culture. However, discrepancies in some expected fluxes and potential perturbation of the system during addition of labelled substrate warrant further development of the method. Future application of INST-MFA to heterotrophic tissues should consider three potential improvements: (i) increasing the number of replicates in order to better quantify the true reliability of the labelling data: (ii) minimising perturbation of the system when adding the labelled substrate; and (iii) parallel labelling experiments with additional positionally labelled substrates. Determining the optimal combination of labelled substrates would require simulation studies based on current best estimates of fluxes and metabolite pool sizes, and this has the potential to increase flux precision and the number of fluxes that can be determined (Nöh and Wiechert, 2006).

### Future prospects for INST-MFA of heterotrophic plant tissues

The motivation for this study was to establish if INST-MFA could quantify fluxes in heterotrophic cells following a metabolic perturbation, where the metabolic steady state may be inaccessible to SS-MFA. The assumption of a pseudo-metabolic steady state is key to both INST-MFA and SS-MFA and, in practical terms, requires the rate of change in the fluxes and intermediate metabolite concentrations to be negligible relative to the time-course of the labelling experiment. INST-MFA labelling time-courses can be captured over relatively short periods of minutes to hours (**Figures 2**, **3**) and therefore can provide access to metabolic systems that are changing more rapidly in comparison to those accessible to SS-MFA. The critical question is whether the system being analysed is changing sufficiently slowly for this assumption to be valid. In this study a new metabolic steady state was assumed to be reached 6 h after addition of menadione based on previous metabolomics analysis of arabidopsis cell cultures (Baxter et al., 2007). In principle, support for this assumption could be provided by measuring the output fluxes of the system. The major output in these heterotrophic cell cultures is new cell biomass, and typically a biomass equation with a fixed stoichiometry based on the relative proportions of compounds that make up the biomass is used to define the output fluxes of the system. However, testing the rate of biomass production and whether its composition is also changing over a short period of time during the labelling experiments is likely to be difficult because it would rely on measuring small changes in the amounts of abundant metabolic end products.

Ultimately, future applications of INST-MFA to heterotrophic plant systems will rely on the ability to test the assumption of a metabolic steady state. The difficulty in confirming this over short time periods following a metabolic perturbation limits the applicability of INST-MFA. Additionally, heterotrophic tissues can be comprised of multiple cell types with distinct metabolic phenotypes. Although reporter proteins can potentially be used in SS-MFA to resolve fluxes in heterogenous tissues (Rossi et al., 2017), INST-MFA is not amenable to the same methods as time-courses are too short to accumulate substantial amounts of label into cell-type specific end products used to differentiate labelling in different cell types. Despite these challenges, recent improvements in crop yields highlight the potential benefits of efficient metabolic adaptation to dynamic conditions (De Souza et al., 2022) and therefore, the ability to accurately analyse fluxes in dynamic or perturbed systems, in both heterotrophic and autotropic tissues, remains an important goal for the success of metabolic engineering in real world conditions.

## Data availability statement

The original contributions presented in the study are included in the article/**Supplementary material**. Further inquiries can be directed to the corresponding author.

## Author contributions

ENS, NJK, and RGR conceived and designed the experiments. ENS performed all the experiments and analysed the data. ENS, NJK, and RGR wrote the paper. ENS is the author responsible for the distribution of materials integral to the findings presented in this article. All authors contributed to the article and approved the submitted version.
