# Supplementary material for: Isotopically non-stationary metabolic flux analysis of heterotrophic Arabidopsis thaliana cell cultures
Source: Front Plant Sci. 2023 Jan 9;13:1049559. doi: 10.3389/fpls.2022.1049559 (PMC9868915; doi:10.3389/fpls.2022.1049559)
Supplement: Supplementary file 1 [file DataSheet_1.docx]

Supplementary Figures and Tables

# Media glucose labelling

Table S1. Proportion of [^13^C_6_]glucose in heterotrophic *Arabidopsis* cell cultures after addition of [^13^C_6_]glucose.

|  | **[^13^C_6_]glucose in medium (%)** | |
| --- | --- | --- |
| **Replicate** | **Control** | **Menadione** |
| A | 62.5 | 59.8 |
| B | 62.8 | 60.6 |
| C | 65.3 | 66.1 |

# MIDs of central carbon metabolites


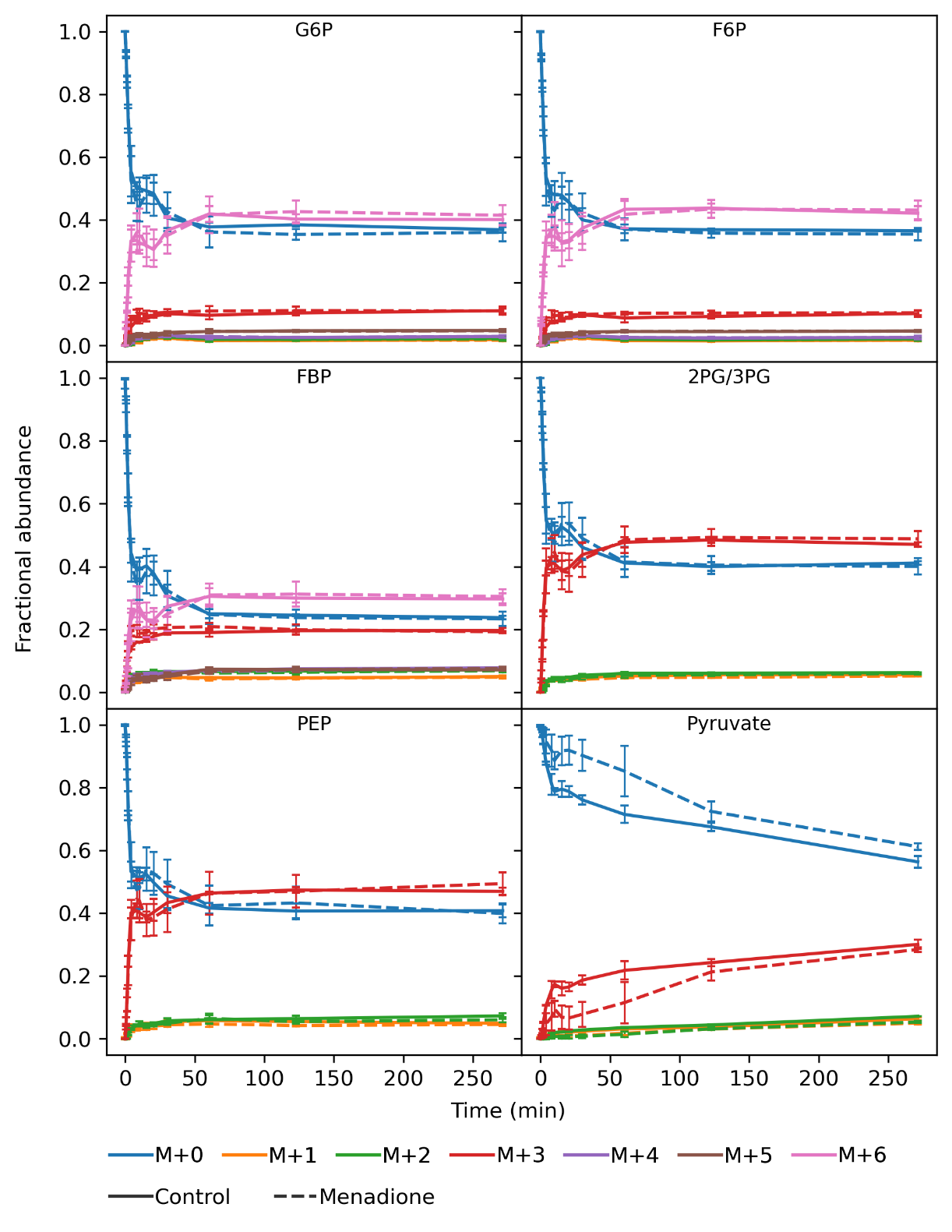


Figure S1. Mass isotopologue distributions of glycolytic intermediates after supply of [^13^C_6_]glucose to heterotrophic Arabidopsis cell cultures following 6 h treatment with 60 µM menadione. MID’s are corrected for natural abundance of heavy isotopes. Values are the mean ± SD n = 3.


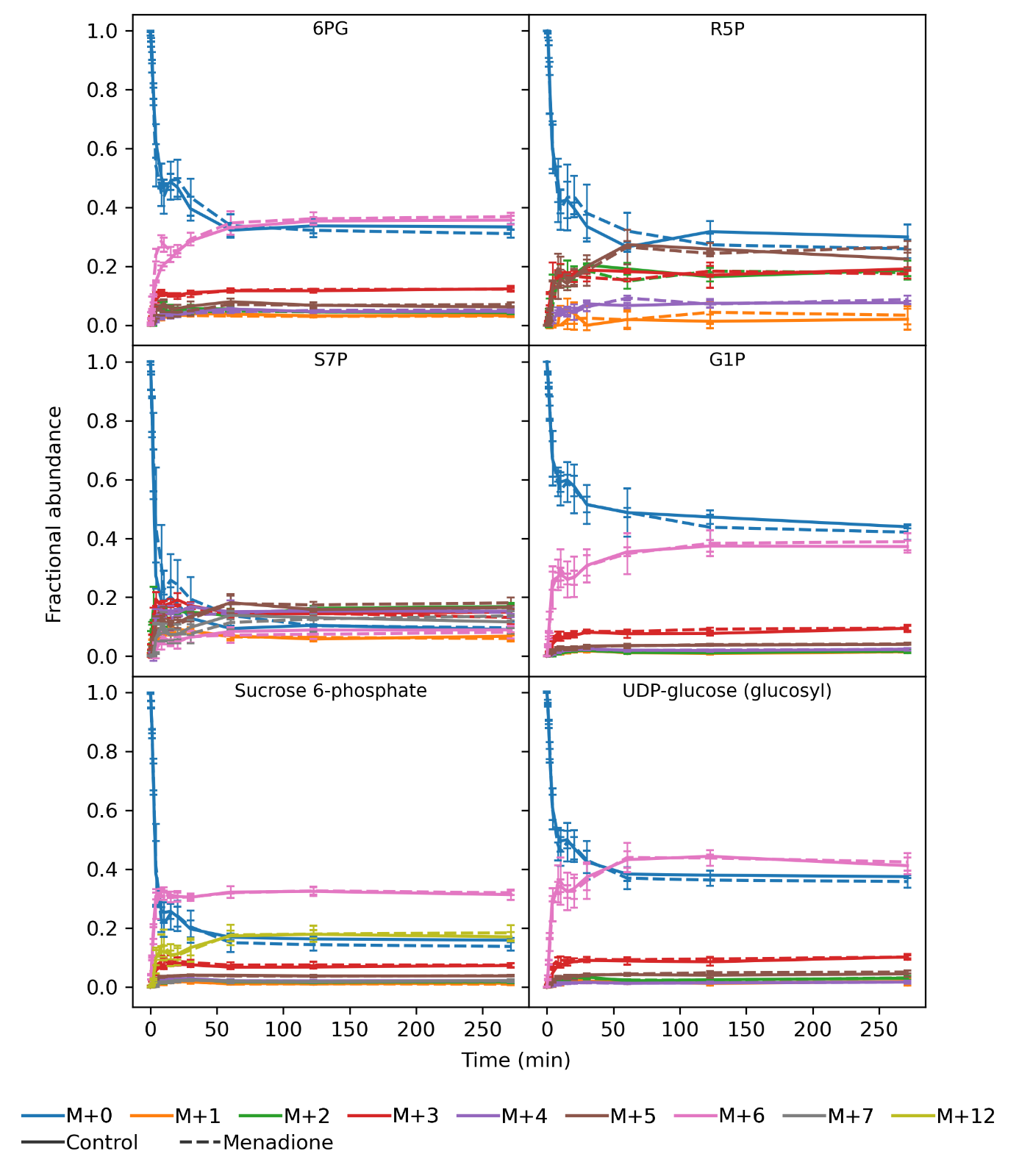


Figure S2. Mass isotopologue distributions of pentose phosphate pathway and starch/sucrose biosynthesis intermediates after supply of [^13^C_6_]glucose to heterotrophic Arabidopsis cell cultures following 6 h treatment with 60 µM menadione. MID’s are corrected for natural abundance. Values are the mean ± SD n = 3.


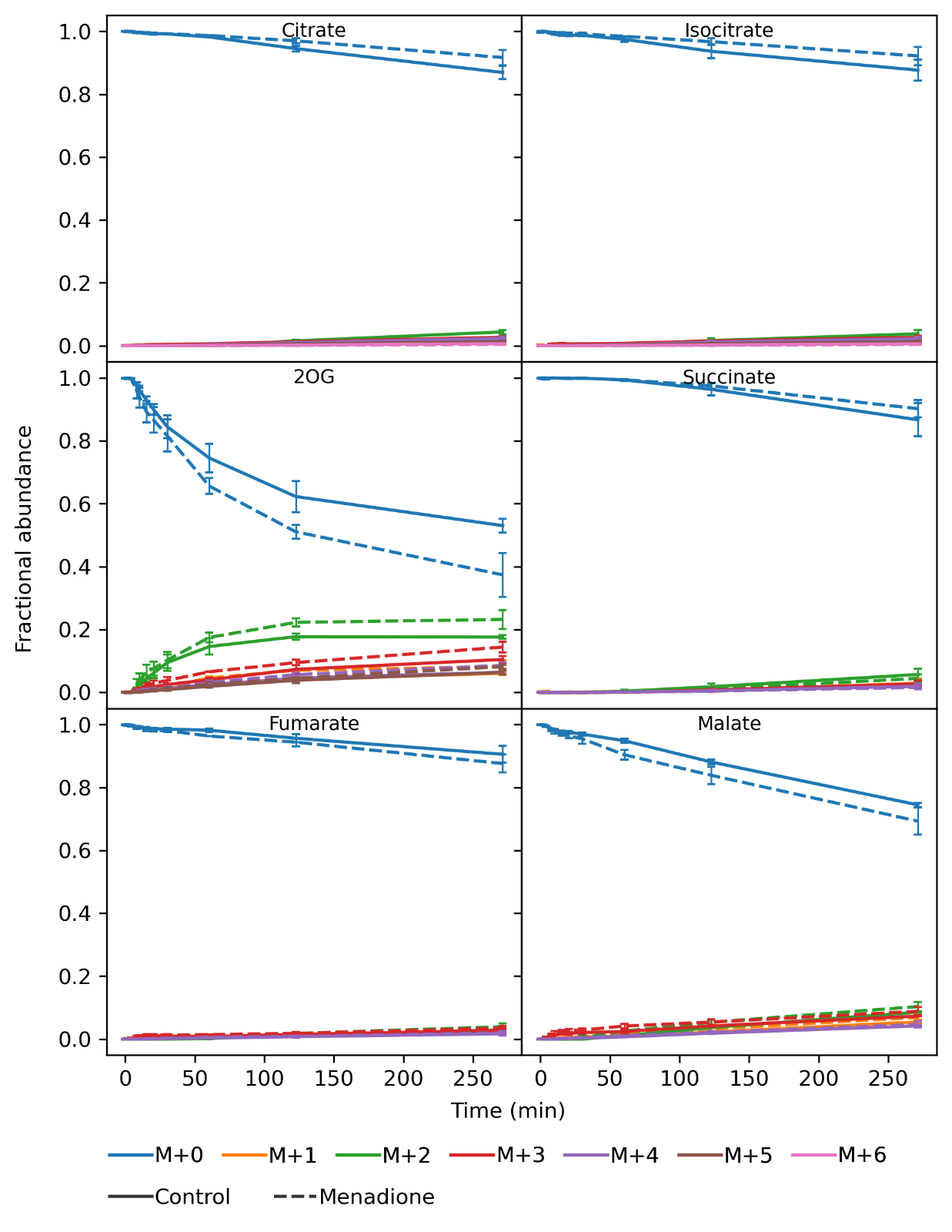


Figure S3. Mass isotopologue distributions of TCA cycle intermediates after supply of [^13^C_6_]glucose to heterotrophic Arabidopsis cell cultures following 6 h treatment with 60 µM menadione. MID’s are corrected for natural abundance. Values are the mean ± SD n = 3.

# The effect of delay in arrival of labelled substrate


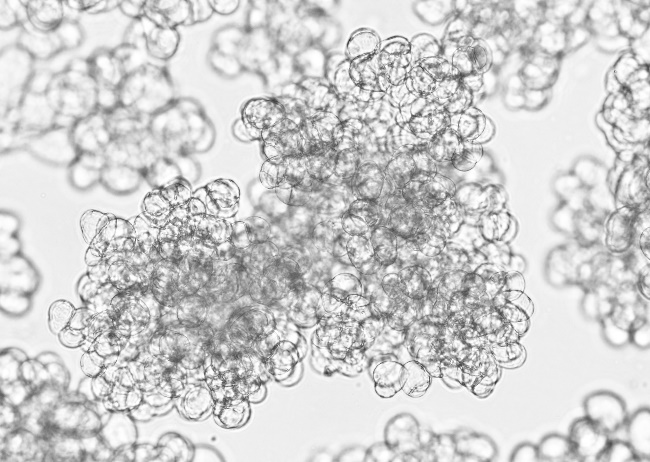
It was observed that the heterotrophic *Arabidopsis* cells clumped together (Figure S4). To explore the potential impact of this on flux estimation by INST-MFA, the time-course of labelled substrate uptake measured from samples of a heterogeneous cell cultures was modelled (Figure S5A) and the simulated data used for flux estimation (Figure S5B).

Figure S4. 4-d-old heterotrophic arabidopsis cell culture imaged using brightfield microscopy.

Figure S.5A shows the exponential decay in the unlabelled fraction of a labelled substrate imported into cells form the media. Clumping of cells may result in a delay in the time taken for the labelled substrate to reach cells in the centre of the clump. The resulting labelling timecourse, measured upon extraction of a metabolite from the population of cells, is a weighted average of these delayed labelling timecourses based on the size of each population of cells. The weighting depends on the structure of the groups of cells. For example, if the clumps of cells approximated to spheres (Figure S5A dashed line) then the size of each population would decrease towards the centre of the sphere as the radius decreases with the relationship 4πr^2^. The delayed labelling timecourse had a sigmoidal shape in contrast to the exponential decay expected in a homogenous cell population (Figure S5A).

To quantify the effect of a delay in the uptake of labelled substrate on the fitting procedure of INST-MFA, an idealised dataset was simulated with no measurement error and delays in labelled glucose uptake from 0 – 30 min simulated based on equal weightings or a spherical distribution of cells. Fluxes were then estimated based on the modified dataset and the sum of squared residuals (SSR) between the simulated measurements and the flux model were calculated (Figure S.5B). Delayed label incorporation in a heterogenous population of cells increased the residual of flux estimation (Figure S.5B). Assuming cells are clustered in spheres, a delay of up to 5 min causes a minimal increase in the SSR (<10). Delays of up to 30 min still cause relatively small increases in the SSR (<500). Therefore, short delays in glucose uptake are unlikely to cause a lack of fit in flux estimations.

Figure S5. Effect of cell culture heterogeneity and delayed substrate uptake on flux analysis. (A) Simulated effect of a heterogenous cell culture on the labelling of cellular metabolites. Eleven populations of cells were simulated with a delay in the time taken for the labelled substrate to reach the cells (grey lines). The average isotopic enrichment from metabolites extracted from the cells is the weighted average of the individual populations assuming there are an equal number of cells in each population (solid black line), or cells are arranged in a 3D sphere where the majority of cells are on the outside and the size of each population decreases with the distribution 4πr^2^ (dashed black line). (B) The effect of a heterogenous cell population causing delayed labelled glucose uptake on flux estimation. An idealised dataset of metabolite labelling time-courses with no delay in labelled substrate uptake was initially simulated. Increasing amounts of delay in labelled glucose uptake were added to the simulated data representing a heterogenous population of cells. Fluxes were estimated using the simulated data and the sum of squared residuals (SSR) calculated using INCA.

# The effect of measurement error on model fitting

The effect of measurement error on model fitting was explored by adjusting the measurement error of the experimental data to a fixed value across different timepoints and metabolites and calculating the SSR after flux estimation (Figure S6). As measurement error was increased the total residuum decreased (Figure S6). A larger error was required to achieve an accepted fit for menadione-treated cells compared to control cells, reflecting the larger variation in the measurements between the three biological replicates in the menadione-treated cells. Differences in the measurement error of individual mass isotopologues and time points from biological replicates can cause overly tight fitting of free parameters to specific time points or isotopologues, as the residual for each measurement is weighted by its standard error. Therefore, defining consistent errors across time points and isotopologues avoided biasing the fitting procedure to a small number of measurements and allowed a statistically acceptable fit between the data and the free parameters to be achieved.

Figure S6. The effect of measurement error on flux estimation residual for control (black circles) and menadione (pink squares) cell cultures. Dashed lines represent the maximum SSR required for an accepted fit. Mol% errors were set to a minimum value for MIDs ≤ 0.5mol% and scaled linearly up to a maximum error for MIDs ≥ 25 mol% (Young, 2014). The maximum error was set 0.7 mol% larger than the minimum error. X-axis is the minimum error value defined for MIDs ≤ 0.5 mol%. SSR – sum of squared residuals.

# Flux map statistical analysis

To identify statistically significant differences in flux distributions, Monte Carlo simulations were performed based on the global best fit flux maps. Multiple estimates for each flux were generated by adding Gaussian noise to each measurement based on the measurement error and then re-fitting the fluxes. Two approaches were used to identify significantly different fluxes. Overlap between 83.4% confidence intervals and multivariate statistics using principal component analyses (PCA) and partial least squares discriminant analysis (PLSDA). PCA and PLSDA were performed on the free net fluxes to identify whether the flux distributions could be separated in control and menadione-treated cells and to identify any outliers (Figure S7).
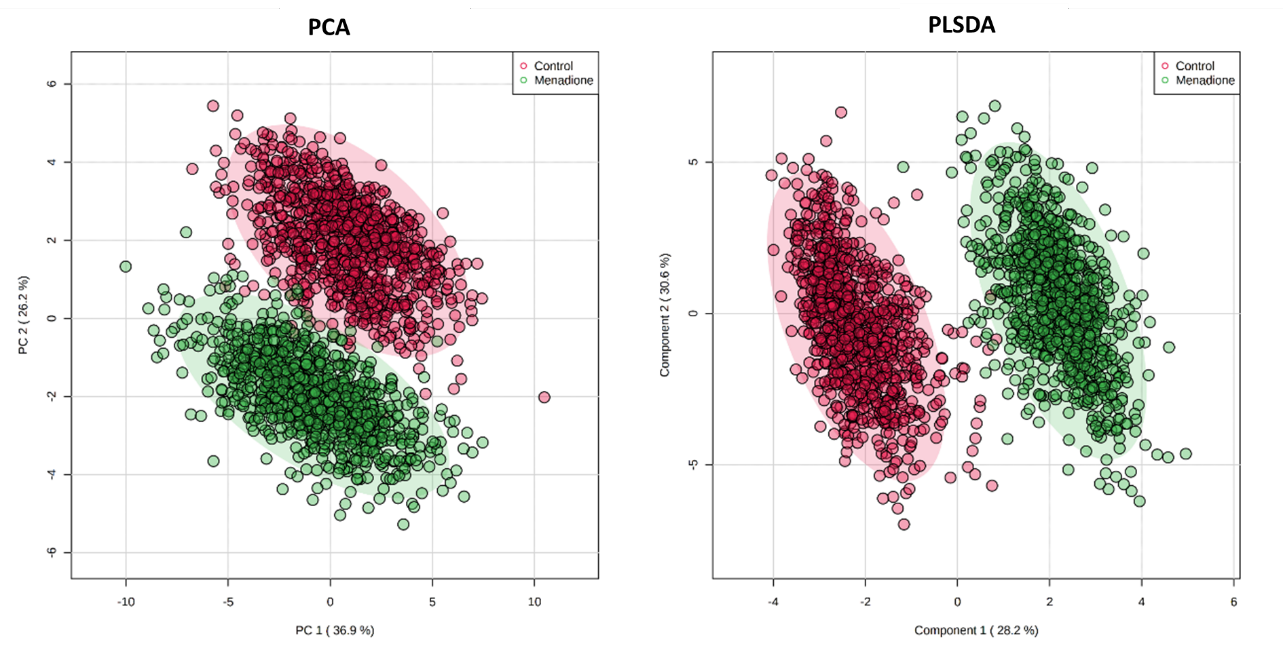


Figure S7. Principal component analysis (PCA) and partial least squares discriminant analysis (PLSDA) of 1000 Monte Carlo simulations of fluxes from control (red) and menadione-treated (green) cell cultures. Fluxes were mean centred and scaled to unit variance. Shaded regions represent 95% confidence interval. Values in brackets on the axis describe proportion of the variation in the data described by the top two principal components. Only free, net fluxes of central carbon metabolism were included in the analysis. Biomass output fluxes, exchange fluxes, pool sizes and pseudo fluxes for mixing of compartmented or unlabelled pools were excluded.

Figure S7 shows that PCA could differentiate between the fluxes in control and menadione treated cells and none of the Monte Carlo simulations are obvious outliers. PSLDA showed clear separation between the groups, as expected from the PCA. To identify which fluxes contributed to the PLSDA separation, variable importance projection (VIP) scores were ranked for each free net flux parameter (Figure S8).


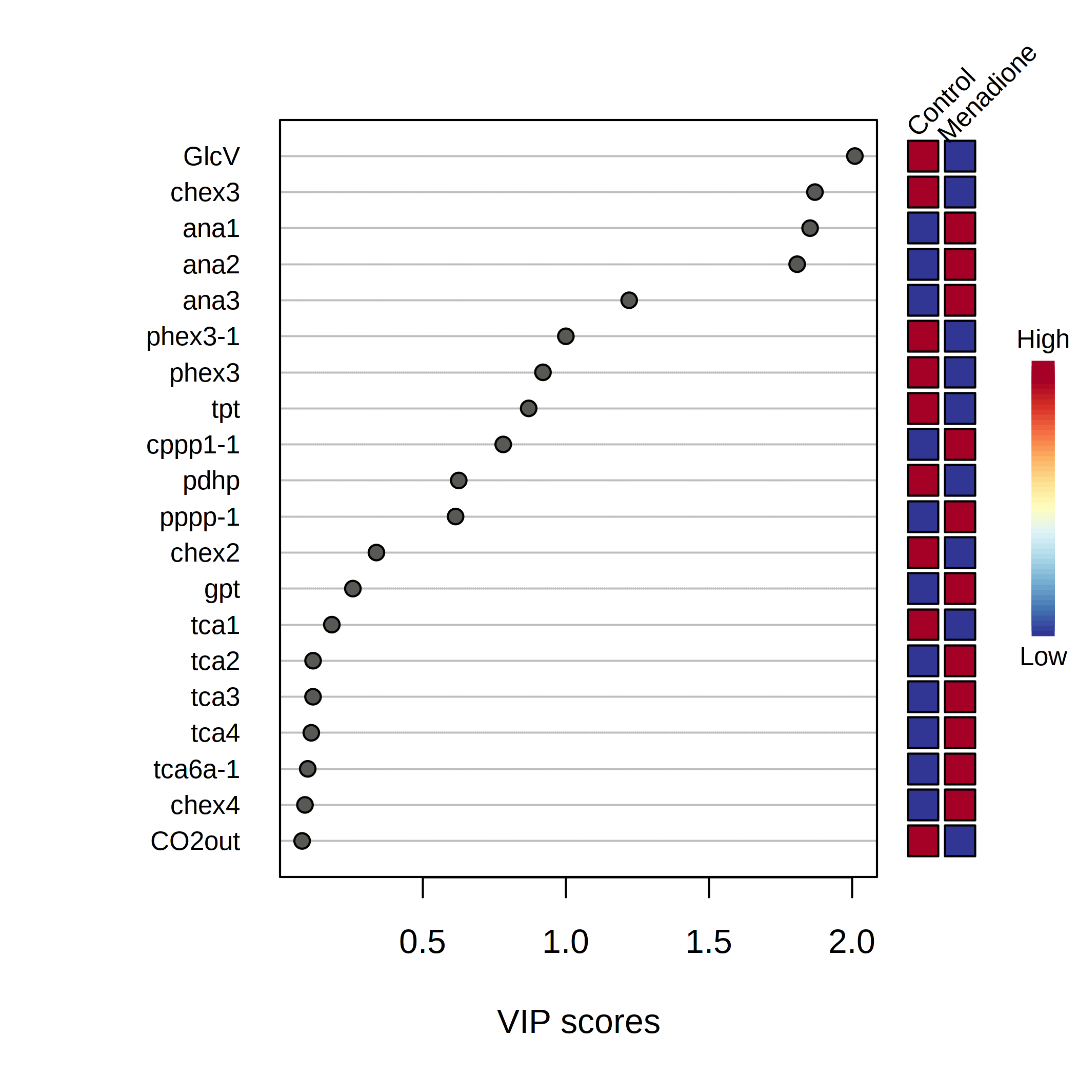


Figure S8 Variable importance in projection (VIP) scores from PLSDA analysis of 1000 Monte Carlo simulations of fluxes from control and menadione-treated cell cultures. Fluxes were mean centred and scaled to unit variance. Only, net fluxes of central carbon metabolism were included in the analysis. Biomass output fluxes, exchange fluxes, pool sizes and pseudo fluxes for mixing of compartmented or unlabelled pools were excluded.

VIP scores identify GlcV, chex3, ana1 and ana2 as making the largest contributions to distinguishing between flux distributions between control and menadione-treated cells, in agreement with comparison of the confidence intervals. TCA cycle fluxes and CO_2_ efflux do not contribute appreciably to separation of the groups confirming these fluxes were not significantly affected by the menadione treatment.

To ensure the PLSDA model was not overfitting the data a cross-validation (Figure S9) and permutation testing (Figure S10) were performed to quantify the effectiveness of the PLSDA model at predicting the group assignment of samples.


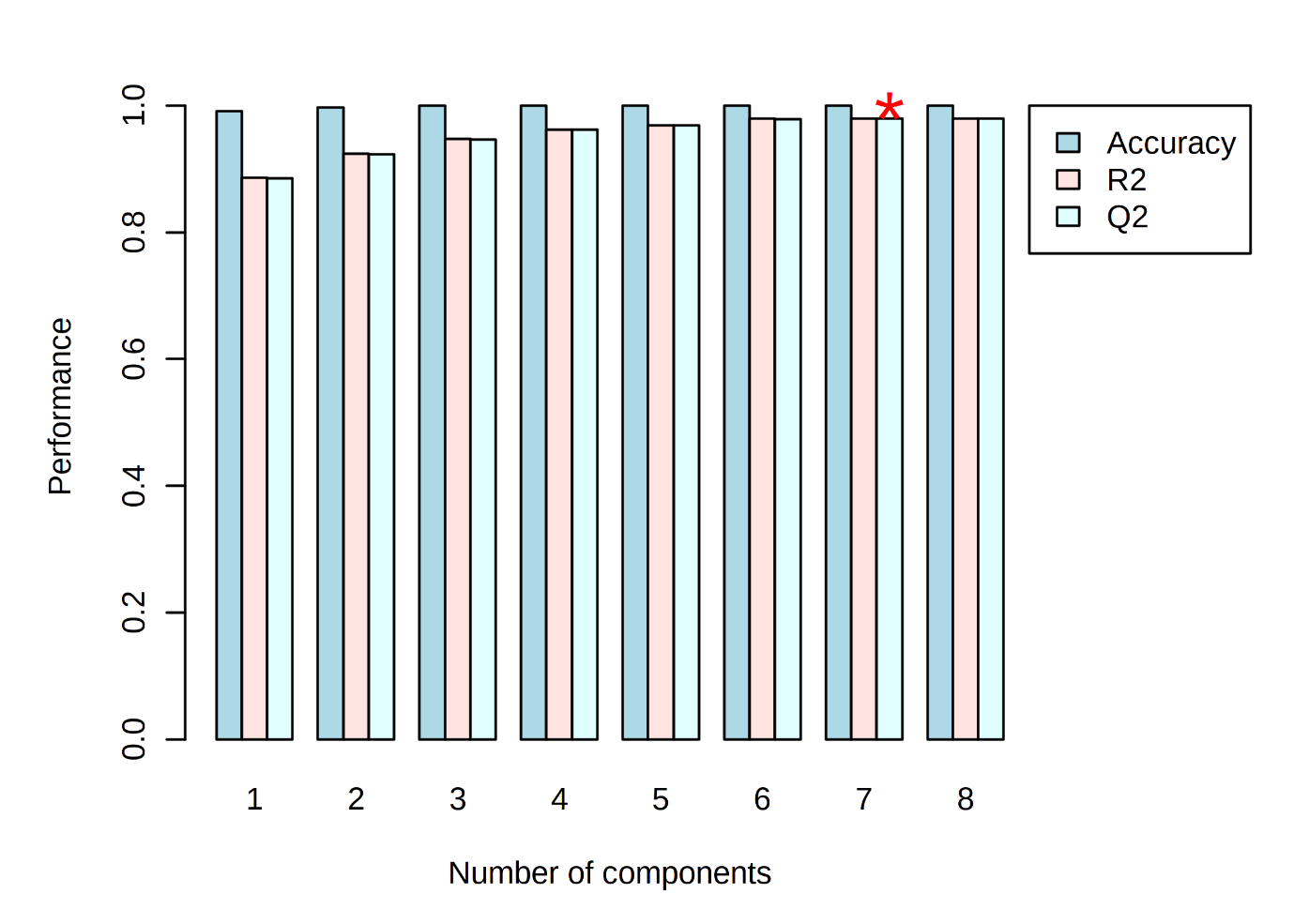


Figure S9. Validation testing of PLSDA analysis by leave-one-out cross-validation. Performance measures included the prediction accuracy (Accuracy), sum of squares captured by the model (R^2^) and the cross-validated R^2^ (Q^2^). The optimal number of components is indicated by (*).

Cross-validation was used identify the optimal number of components required for the PLSDA model (Westerhuis et al., 2008; Szymańska et al., 2012). In each cross-validation the predicted data was compared with the original data and sum of squared errors calculated. Assessment of the performance measures showed that the optimum model contained seven-components and displayed 100% accuracy with R^2^ (0.98) and Q^2^ (0.98) (Figure S9).

To confirm the statistical significance of the performance measures, permutation testing was performed to compare the trained model to a model generated with randomly permuted data (Figure S10). In each permutation the labels of the samples were randomly assigned, and a new classification model was calculated using the optimal number of components determined previously (Figure S9). The trained classification model outperformed the family of randomly permuted models establishing that the identified difference between the menadione and control samples was statistically significant (Figure S10).


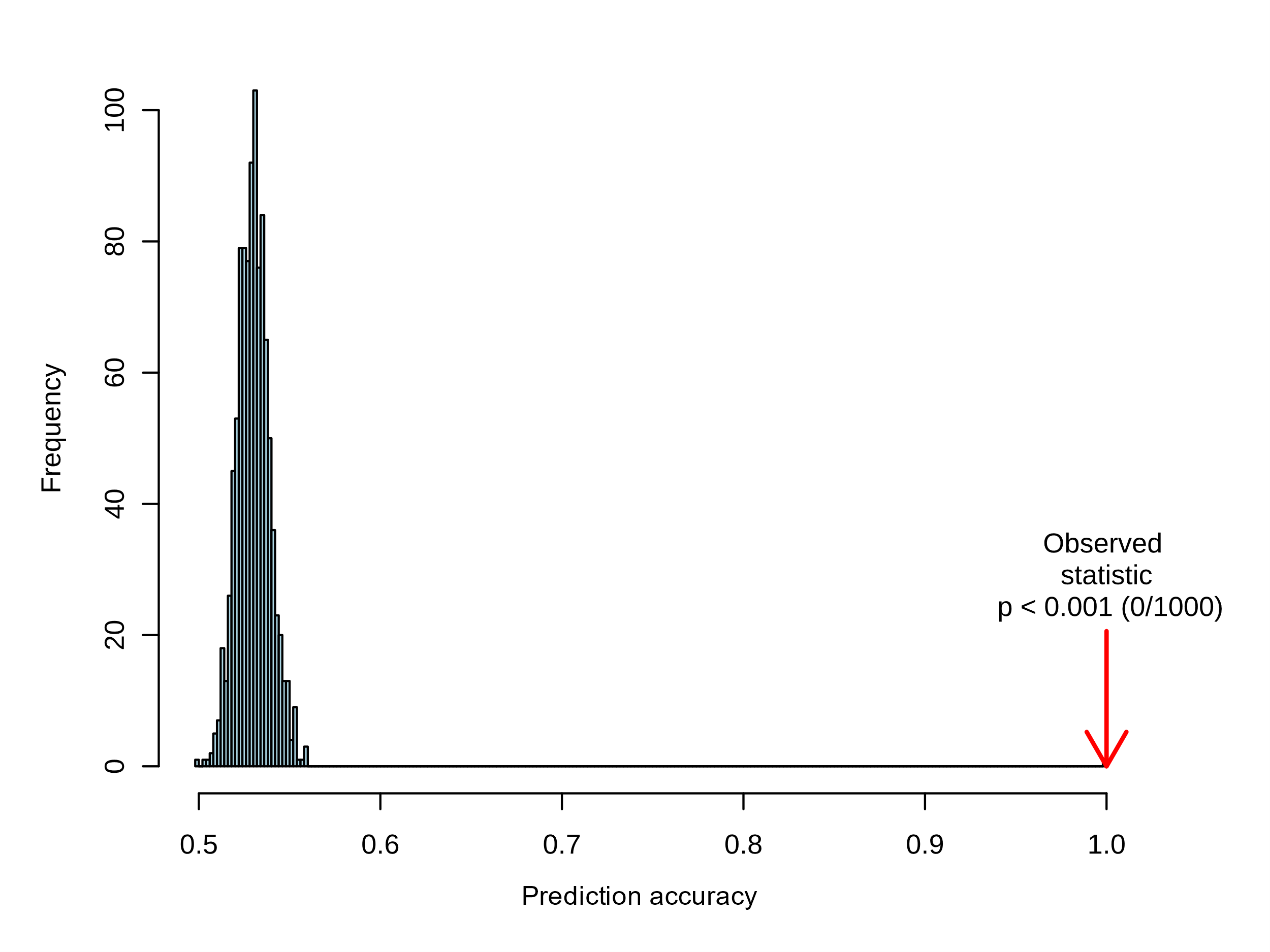


Figure S10. Prediction accuracy of randomly permutation PLSDA model (histogram, blue bars) compared to the optimal trained model (red arrow). None of the randomly permutated models outperformed the original model (p < 0.001 0/1000).

# References

Szymańska, E., Saccenti, E., Smilde, A. K., and Westerhuis, J. A. (2012). Double-check: Validation of diagnostic statistics for PLS-DA models in metabolomics studies. *Metabolomics* 8, 3–16. doi: 10.1007/s11306-011-0330-3.

Westerhuis, J. A., Hoefsloot, H. C. J., Smit, S., Vis, D. J., Smilde, A. K., Velzen, E. J. J., et al. (2008). Assessment of PLSDA cross validation. *Metabolomics* 4, 81–89. doi: 10.1007/s11306-007-0099-6.

Young, J. D. (2014). INCA: A computational platform for isotopically non-stationary metabolic flux analysis. *Bioinformatics* 30, 1333–1335. doi: 10.1093/bioinformatics/btu015.
